# Supplementary material for: Empirical methods for controlling false positives and estimating confidence in ChIP-Seq peaks
Source: BMC Bioinformatics. 2008 Dec 5;9:523. doi: 10.1186/1471-2105-9-523 (PMC2628906; doi:10.1186/1471-2105-9-523)
Supplement: Additional File 1 — A variety of html documents from the USeq web site detailing the available applications, their best usage, output file type descriptions, command line menus, etc. [file 1471-2105-9-523-S1.zip › USeqUserGuides/JavaDoc.zip!/JavaDoc/expr/NormalizeAgilentData.html]

NormalizeAgilentData


|  |  |  |
| --- | --- | --- |
| **Overview** \| **Package** \| **Class** \| **Use** \| **Tree** \| **Deprecated** \| **Index** \| **Help** | |  |
| **PREV CLASS**   **NEXT CLASS** | **FRAMES**    **NO FRAMES**     **All Classes** |  |
| SUMMARY: NESTED \| FIELD \| CONSTR \| METHOD | DETAIL: FIELD \| CONSTR \| METHOD |  |


---


## expr Class NormalizeAgilentData

```
java.lang.Object
  expr.NormalizeAgilentData
```

---

``` public class NormalizeAgilentData extends java.lang.Object ```

Takes two replicas, parses out those probes that are not 1's, median centers, then averages
ProbeName ProbeName LogRatio gIsPosAndSignif rIsPosAndSignif LogRatio gIsPosAndSignif rIsPosAndSignif
A\_51\_P100021 A\_51\_P100021 -0.087965 1 1 0.344989 0 1
A\_51\_P100034 A\_51\_P100034 0.038444 1 1 0.034538 1 1
A\_51\_P100052 A\_51\_P100052 -0.146955 1 0 0 0 0

---

**Constructor Summary**

`NormalizeAgilentData(java.lang.String[] args)`


| **Method Summary** | |
| --- | --- |
| `static void` | `main(java.lang.String[] args)` |

**Methods inherited from class java.lang.Object**

`equals, getClass, hashCode, notify, notifyAll, toString, wait, wait, wait`

**Constructor Detail**

### NormalizeAgilentData

```
public NormalizeAgilentData(java.lang.String[] args)
```


**Method Detail**

### main

```
public static void main(java.lang.String[] args)
```


---


|  |  |  |
| --- | --- | --- |
| **Overview** \| **Package** \| **Class** \| **Use** \| **Tree** \| **Deprecated** \| **Index** \| **Help** | |  |
| **PREV CLASS**   **NEXT CLASS** | **FRAMES**    **NO FRAMES**     **All Classes** |  |
| SUMMARY: NESTED \| FIELD \| CONSTR \| METHOD | DETAIL: FIELD \| CONSTR \| METHOD |  |


---
